# Specific profile of ultrasonic communication in a mouse model of neurodevelopmental disorders

**DOI:** 10.1038/s41598-019-52378-0

**Published:** 2019-11-04

**Authors:** Marika Premoli, Sara Anna Bonini, Andrea Mastinu, Giuseppina Maccarinelli, Francesca Aria, Giulia Paiardi, Maurizio Memo

**Affiliations:** Department of Molecular and Translational Medicine, University of Brescia, Viale Europa 11, 25123 Brescia, Italy

**Keywords:** Neuroscience, Developmental biology

## Abstract

Mice emit ultrasonic vocalizations (USVs) in different social conditions: pups maternal separation, juveniles play, adults mating and social investigation. The USVs measurement has become an important instrument for behavioural phenotyping in neurodevelopmental disorders (NDDs). Recently, we have demonstrated that the deletion of the NFκB1 gene, which encodes the p50 NF-κB subunit, causes NDDs phenotype in mice. In this study, we investigated the ultrasonic communication and the effects of an early social enrichment in mice lacking the NF-κB p50 subunit (p50 KO). In particular, USVs of wild-type (WT), p50 KO and KO exposed to early social enrichment (KO enriched) were recorded using an ultrasound sensitive microphone and analysed by Avisoft software. USVs analysis showed that p50 KO pups emit more and longer vocalizations compared to WT pups. On the contrary, in adulthood, p50 KO mice emit less USVs than WT mice. We also found significant qualitative differences in p50 KO mice USVs compared to WT mice; the changes specifically involved two USVs categories. Early social enrichment had no effect on USVs number, duration and type in p50 KO mice. Together, these data revealed social communication alterations in a mouse model of NDDs; these deficits were not recovered by early social enrichment, strengthening the fact that genetic background prevails on environmental enrichment.

## Introduction

Mice communicate each other in the ultrasonic range of sound frequencies, above the limit of human hearing^1^. They emit ultrasonic vocalizations (USVs) in different social conditions: pups when separated from the littermates and the mother^2,3^, juveniles in social play and adults during courtship, mating and social interaction^4,5^.

Since the early 1970s, the functional role of USVs has been extensively debated. These sounds emitted by pups were initially described as a simple by-product of a physiological response to a thermal challenge^6,7^. Subsequently, other researchers demonstrated that USVs can have a communicative purpose and can express an emotional state^8^. In particular, USVs of pups elicit maternal care and they are interpreted as an early communicative signal of mother-pup interaction^2,9^. Lahvis confirmed emotional information of USVs and suggested that variations in the mouse vocal repertoire can indicate changes in affective states of mice^10^. Also adult mouse USVs are signals of internal emotional states and facilitate social communication during non-aggressive encounters, and in particular during mating.

As communicative signals, USVs play an important role in the study of the genetic basis of neurodevelopmental disorders (NDDs)^11^. Mice ultrasonic communication analysis has become an important tool for behavioural readout and monitoring^12,13^. Altered calling patterns are detected in different mouse models of NDDs, especially in models of autism spectrum disorders (ASD); for example, BTBR T + tf/J^14,15^ and Fmr1-KO (knock-out) mice^16–19^ showed quantitative and qualitative alterations from their wild-type controls (WT) during infancy and in adulthood. Ultrasonic communication deficits were also found in other mouse models of ASD, such as the Shank mouse lines^20–22^ and Ctnap2 KO mice^23^. Similar results were obtained from the characterization of models associated with NDDs such as neuroligins KO mice^24,25^, oxytocin receptor KO mice^26^, Tph2 null mutant mice^27^ and 16p11.2 deletion mice^28^.

USVs have been largely studied in a rodent ethological perspective in the last decades, but only recently the importance of the study of USVs in the context of NDDs is emerged. Moreover, the analysis of ultrasonic communication in animal models of NDDs is also interesting for its translational value with patients affected by NDDs. Indeed, USVs in mice can model baby cries in the context of ASD^13,14,29^.

Several studies have shown that environmental enrichment (EE) can revert and/or prevent pathological conditions linked to genetic alterations and environmental insults^30^. Given that the mother represents the main component of the environment of a mouse pup, enhancing maternal care constitutes the earliest source of EE. This stimulation of mother-pup interactions can be obtained by housing pups with an additional female from birth until weaning^31^. This early enrichment has been reported to have long-term beneficial effects on brain and behaviour in WT mice and more importantly in murine models of NDDs^31,32^.

Based on this context, we analysed ultrasonic communication in another mouse model of NDDs, p50 KO mice that present a deletion of the NFκB1 gene, coding for the NF-κB p50 subunit. p50 is a member of the NF-κB family. In response to numerous stimuli, NF-κB is rapidly activated and plays different roles in many cellular processes including regulation of neuronal survival and plasticity, inflammation, cell proliferation, neurogenesis, apoptosis, oncogenesis, learning and memory^33–39^. After six months of age p50 KO mice have a reduced body weight in comparison with WT and display a particular behaviour characterized by decreased anxiety-like responses, increased exploratory activity and reduced tendency to establish dominant-subordinate relationships among cage mates^40^. They also present altered hippocampal neurogenesis linked to a cognitive deficit in spatial short-term memory^41^. Furthermore, we have previously demonstrated that p50 KO mice have cortical structure abnormalities and social behaviour impairment typical of NDDs animal models^42,43^. Nonetheless, it is still unknown whether p50 KO mice have quantitative and/or qualitative impairments in communication. Hence, in this study we investigated the ultrasonic communication and evaluated the effects of early social enrichment in p50 KO mice on USV emission both in infancy and adulthood. Ultrasonic communication analyses were associated in infancy with measures of neurodevelopment (e.g. eyes opening) and of social interest (homing test), in adolescence with evaluation of sensory abilities (olfactory habituation/dishabituation) and at adulthood with measures of locomotion (open field) and social interaction (male-female social interaction).

## Results

### Reduced maternal care in p50 KO mice

**Figure 1:**
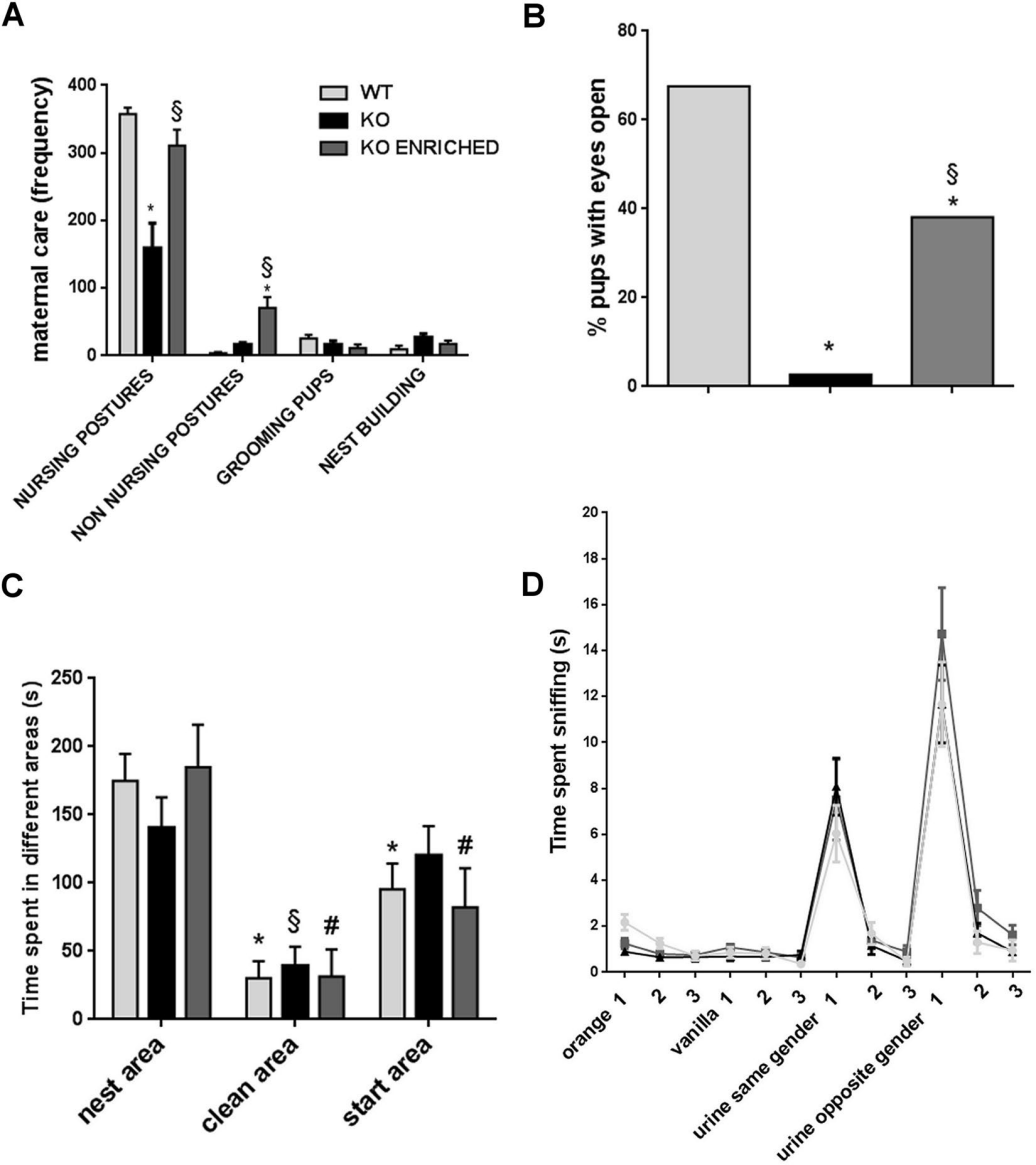
Maternal care assessment and developmental behavioural traits in WT, KO and KO ENRICHED mice. (**A**) Detailed representation of different maternal activities frequency in WT, KO and KO ENRICHED pups from PND 1 to PND 7. Data are presented as mean ± S.E.M. N = 6 WT, 6 KO and 5 KO ENRICHED. *p < 0.05 for KO and KO ENRICHED vs WT pups and §p < 0.05 for KO ENRICHED vs KO pups (Two-way ANOVA, followed by Tukey’s post-test analysis). (**B**) Eyes opening was evaluated in pups on PND 12. N = 37 WT, 37 KO and 21 KO ENRICHED. *p < 0.05 for KO and KO ENRICHED vs WT and §p < 0.05 for KO ENRICHED vs KO mice. (One-way ANOVA, followed by Tukey’s post-test analysis). (**C**) Social recognition was evaluated in the homing test on PND 14 by measuring the time in seconds (s) spent in the nest area, in the clean area and in the start area. Data are presented as mean ± S.E.M. N = 37 WT, 37 KO and 20 KO ENRICHED. *p < 0.05 for time spent in the clean area or start area vs time in nest area for WT; §p < 0.05 for time spent in the start area vs time in nest area for KO; #p < 0.05 for time spent in clean area vs time in nest area for KO ENRICHED and #p < 0.05 for time spent in start area vs time in nest area for KO ENRICHED (Two-way ANOVA, followed by Sidak’s post-test analysis). (**D**) Graphic representation of data collected during olfactory habituation/dishabituation test by measuring the time in seconds (s) spent by adolescent mice in sniffing cotton-tipped swabs saturated with different odours. Data are presented as mean ± S.E.M. N = 20 WT, 20 KO and 17 KO ENRICHED. (Two-way ANOVA, followed by Sidak’s post-test analysis).

Maternal care, such as nursing postures, non nursing postures, pups grooming and nest building, were analysed during the first postnatal week in WT, KO and KO enriched mice (Fig. 1). Maternal behaviour was different between genotypes [F (2, 14) = 15.48, p < 0.001; Fig. 1A]. KO pups received significantly lower nursing postures in comparison with WT pups. On the contrary, KO enriched pups got higher nursing postures than KO pups and similar to those of WT pups. In addition, early social enrichment increased non nursing postures [F (6, 42) = 15.71, p < 0.0001]. Furthermore, it was interesting to assess the individual contribution of the two females to the behaviours measured in social enrichment condition. For this reason, maternal activities of KO mothers and WT females were observed and analysed (Supplementary Fig. S1A). KO mothers displayed more nursing postures and less non nursing postures than WT females [F (3, 24) = 12.71, p < 0.0001]. These data highlight the fact that KO enriched females expressed more nursing postures than when they were alone [F (3, 27) = 2.99, p < 0.05; Supplementary Fig. S1B]. So early social enrichment increased maternal care, but the body weight of pups did not change. Indeed, body weight of KO enriched pups was similar to those of KO pups (Supplementary Fig. S2).

These data consolidate improved maternal care also for p50 KO mice. As reported previously, this could influence neurodevelopment of pups. To verify this, we analysed ultrasonic communication and some neurodevelopment markers in KO and KO enriched compared to WT mice.

### Eyes opening delay in p50 KO and p50 KO enriched pups

Eyes opening, a typical developmental parameter, was evaluated and significant differences between genotypes were found (Fig. 1B) [F (2, 92) = 25.56, p < 0.0001]. On postnatal day (PND) 13 all mice had their eyes open. Differences were found on PND 12 when 68% of WT and only 9% of KO mice opened their eyes. These data suggest developmental delay in KO pups. Interestingly, on PND 12 38% of KO enriched pups presented open eyes, therefore reflecting a heterogeneous phenotype.

On PND 14 social recognition was evaluated in the homing test. No significant genotype effects were found on the latency to reach the area covered by nest sawdust, on locomotor activity (Supplementary Fig. S3) and on the time spent in all different areas (Fig. 1C). Interestingly, pups of all genotypes spent more time in the nest area than in the clean area [F (2, 182) = 19.42, p < 0.0001; Fig. 1C and Supplementary Fig. S3]. WT and KO enriched pups preferred to spend time in the nest area respect to all other areas; instead KO pups spent similar time in the nest and start area. This suggests a partial deficit of social recognition in KO pups, recovered by early social enrichment.

To investigate if results obtained in the homing test depend on any olfactory deficit, olfactory habituation/dishabituation test was performed on adolescent mice (Fig. 1D). There were not significant differences in time spent sniffing odours between genotypes. Data obtained reveal that KO and KO enriched mice have not olfactory deficit.

### Altered ultrasonic communication in p50 KO and p50 KO enriched pups

#### Quantitative analysis of USVs

**Figure 2:**
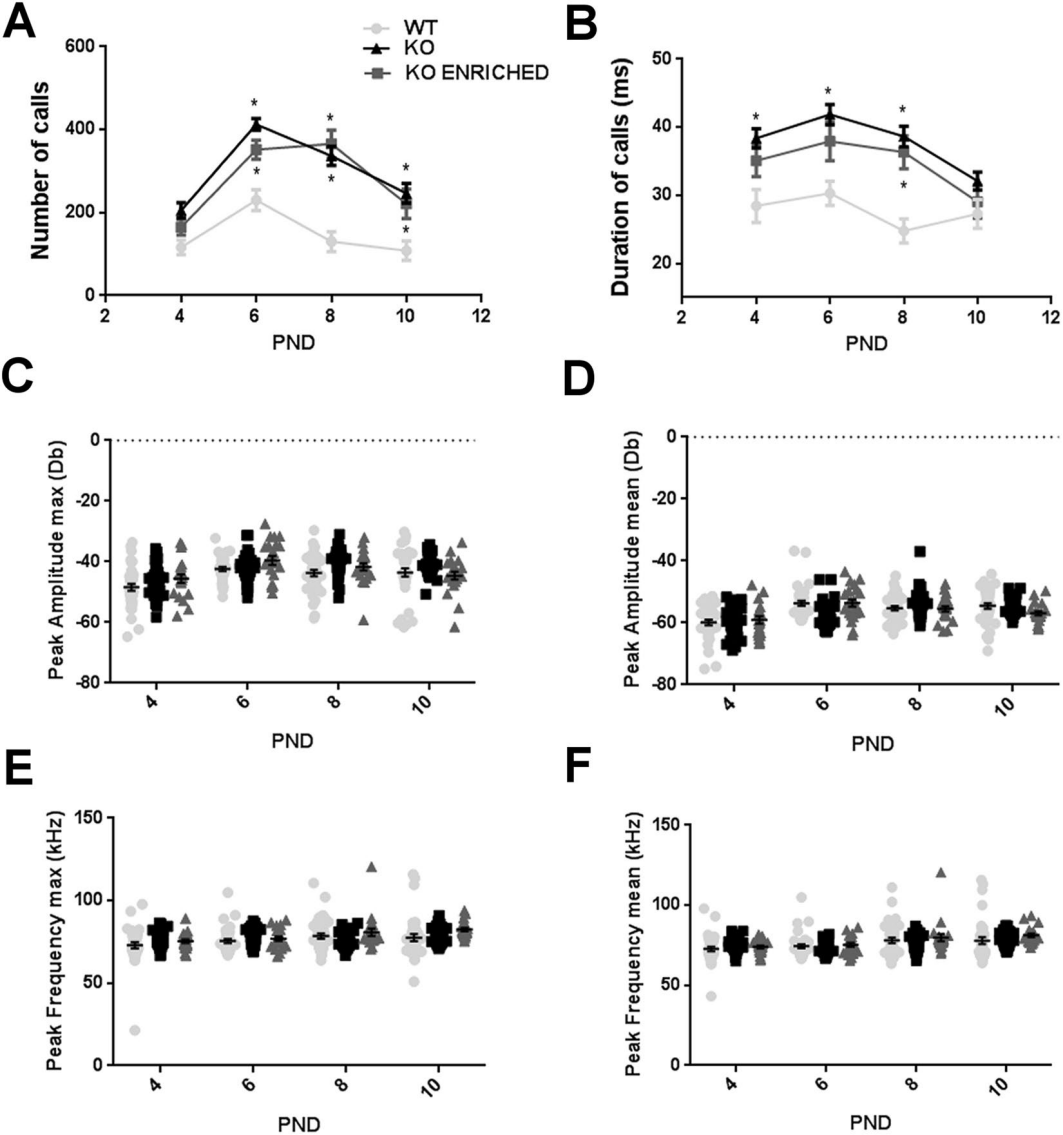
Quantitative analysis of ultrasonic communication in WT, KO and KO ENRICHED pups. (**A**) Number, (**B**) duration, (**C**) peak amplitude max, (**D**) peak amplitude mean, (**E**) peak frequency max and (**F**) peak frequency mean of vocalizations on PND 4, 6, 8 and 10 in response to social separation during a three minute session. Data are presented as mean ± S.E.M. N = 37 WT, 37 KO and 21 KO ENRICHED. *p < 0.05 for KO and KO ENRICHED vs WT pups (Two-way ANOVA, followed by Sidak’s post-test analysis).

For the first time, ultrasonic communication was analysed in this model. WT, KO and KO enriched pups were evaluated on PND 4, 6, 8 and 10 for USVs in response to maternal separation. WT pups emitted a number of USVs that increased from PND 4 to PND 6, when it reached its maximum and then gradually decreased. This pattern was exacerbated in KO. USVs number of KO enriched pups increased from PND 4 to PND 8 and then decreased (Fig. 2A).

Regarding the number of USVs, KO emitted significantly more calls than WT pups [F (2, 92) = 48.19 p < 0.0001; Fig. 2A]. Duration of USVs of KO was significantly longer that those of WT pups [F (2, 92) = 16.68 p < 0.0001; Fig. 2B]. Early social enrichment did not influence ultrasonic communication pattern; indeed USVs of KO enriched were similar to those of KO pups. Concerning other communication traits, such as peak amplitude max and mean, peak frequency max and mean of USVs, no significant differences were found between genotypes (Fig. 2C–F).

These results show altered ultrasonic communication of KO pups, both at basal condition and with early social enrichment. In addition, a more detailed analysis was performed to investigate if there were differences in patterns of calling between female and male pups. No significant differences were detected (Supplementary Figs S4–6).

#### Qualitative analysis of USVs

Considering that highest number of USVs was observed at PND 6 both in WT and KO pups, spectrograms of pups on this day were manually analysed and USVs were classified into 10 categories as previously reported by Scattoni^14^. In Supplementary Fig. S7, some examples of different USVs emitted by WT pups are reported. Also KO and KO enriched pups had the same pattern of classification.

**Figure 3:**
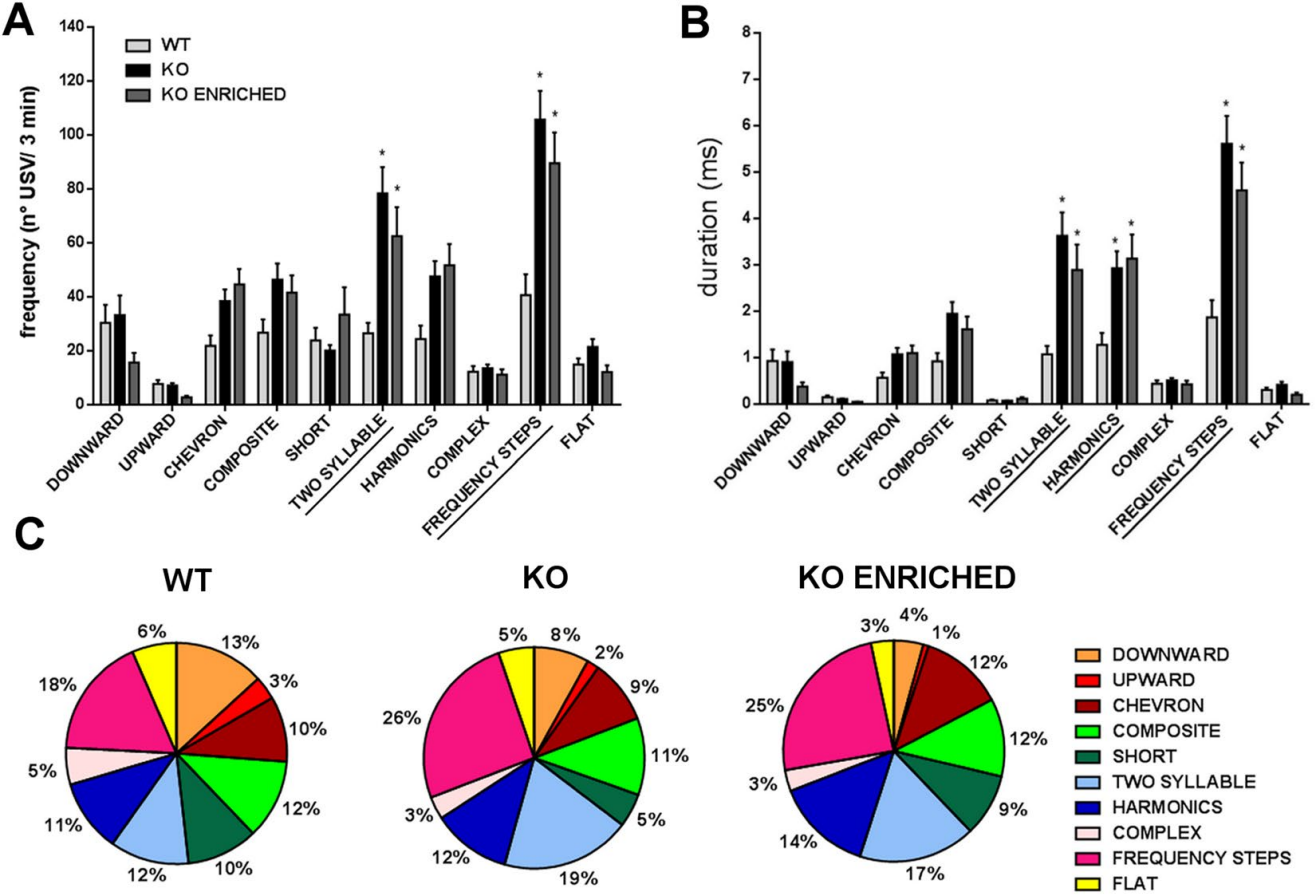
Qualitative analysis of ultrasonic communication in WT, KO and KO ENRICHED pups. (**A**) Frequency (expressed as number of USVs in 3 minutes test) and (**B**) duration of different categories of pups calls at PND 6. Data are presented as mean ± S.E.M. N = 37 WT, 37 KO and 21 KO ENRICHED. *p < 0.05 for KO and KO ENRICHED vs WT pups (Two-way ANOVA, followed by Sidak’s post-test analysis). (**C**) Proportion of different call typologies for genotype expressed as the percentages in pie graphs.

As shown in Fig. 3A-B, both frequency (number of USVs/3 minutes) [F (9, 828) = 40.05, p < 0.0001] and duration of different types of USVs [F (9, 828) = 68.43, p < 0.0001] were different between genotypes. In particular, KO pups emitted a significantly higher number of *two syllable* and *frequency steps* calls in comparison with WT pups [F (18, 828) = 5.760, p < 0.0001]. The duration of *two syllable*, *harmonics* and *frequency steps* of KO pups was higher than WT [F (18, 828) = 8.031, p < 0.0001]. Again, KO enriched pups emitted an ultrasonic communication similar to those of KO pups. This pattern of communication is also shown in Fig. 3C, where the proportions of different call typologies for genotype are represented.

Data obtained confirmed altered ultrasonic communication in KO and KO enriched pups compared to WT pups, also at qualitative level.

### Increased exploration, reduced social interaction and altered communication in p50 KO and p50 KO enriched adult mice

**Figure 4:**
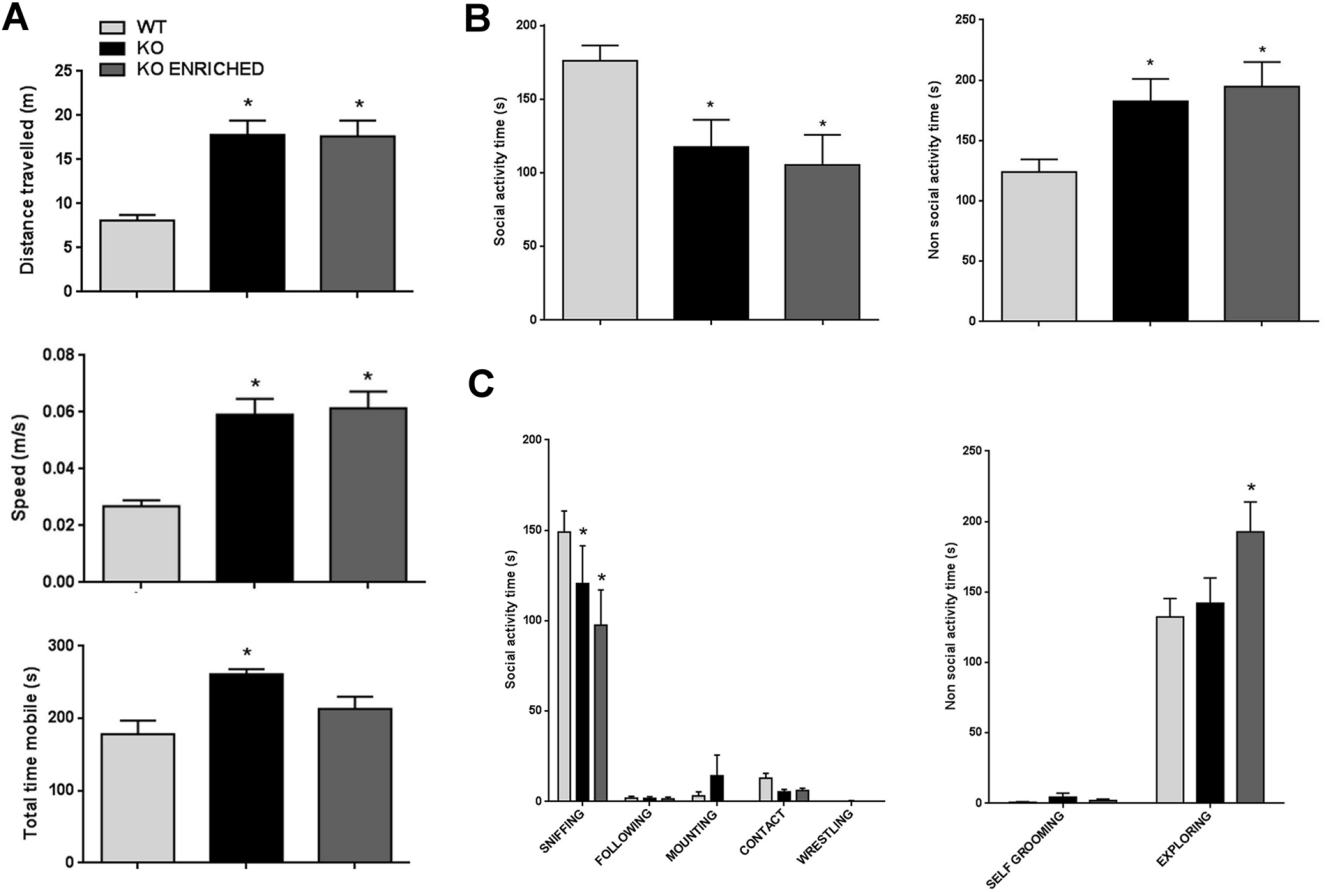
Exploratory and social behaviour of WT, KO and KO ENRICHED adult mice. (**A**) Graphic representation of data collected in the open field test by automatically measuring the total distance travelled, the speed, and the total time mobile. Data are presented as mean ± S.E.M. N = 6 WT, 7 KO and 7 KO ENRICHED. *p < 0.05 for KO and KO ENRICHED vs WT mice (One-way ANOVA, followed by Tukey’s post-test analysis). (**B**) Time spent by WT, KO and KO ENRICHED adult mice in doing social and non-social activities during the male-female social interaction test. Data are presented as mean ± S.E.M. N = 7 couples of mice for genotype. *p < 0.05 for KO and KO ENRICHED vs WT mice (One-way ANOVA, followed by Dunnet’s post-test analysis). (**C**) Detailed representation of time spent doing social activities such as sniffing, following, mounting, contact, wrestling and non social activities as self grooming and exploring. Data are presented as mean ± S.E.M. N = 7 couples of mice for genotype. *p < 0.05 for KO and KO ENRICHED vs WT mice (Two-way ANOVA, followed by Sidak’s post-test analysis).

In order to investigate if alterations found in pups were present also in adult mice, analyses of exploratory activity and social interaction were performed. At first, WT, KO and KO enriched adult males mice were tested in the open field test. As previously demonstrated^42^, KO mice showed increased locomotion and exploratory behaviour in comparison with WT mice [distance travelled: F (2, 17) = 12.66, p < 0.001; speed: F (2, 17) = 13.85, p < 0.001; total time mobile: F (2, 17) = 7.642, p < 0.01; Fig. 4A]. Also KO enriched mice displayed a hyper-activity similar to KO mice (p = 0.99).

To analyse social behaviour, male-female social interaction test was done. As shown in Fig. 4B, KO and KO enriched mice had social interaction deficit. Indeed, KO spent significantly less time in social activities and more time in non social activities than WT mice [F (2, 18) = 4.935, p < 0.05]. Early social enrichment did not ameliorate social deficit. In particular, a more detailed analysis of social and non social behaviours displayed a lower time spent doing sniffing behaviour for KO and KO enriched mice in comparison with WT mice [F _behaviour_ (4, 72) = 114.6, p < 0.0001; Fig. 4C]. In addition, KO enriched spent higher time doing exploring than WT mice [F _behaviour_ (1, 18) = 209, p < 0.0001; Fig. 4C].

**Figure 5:**
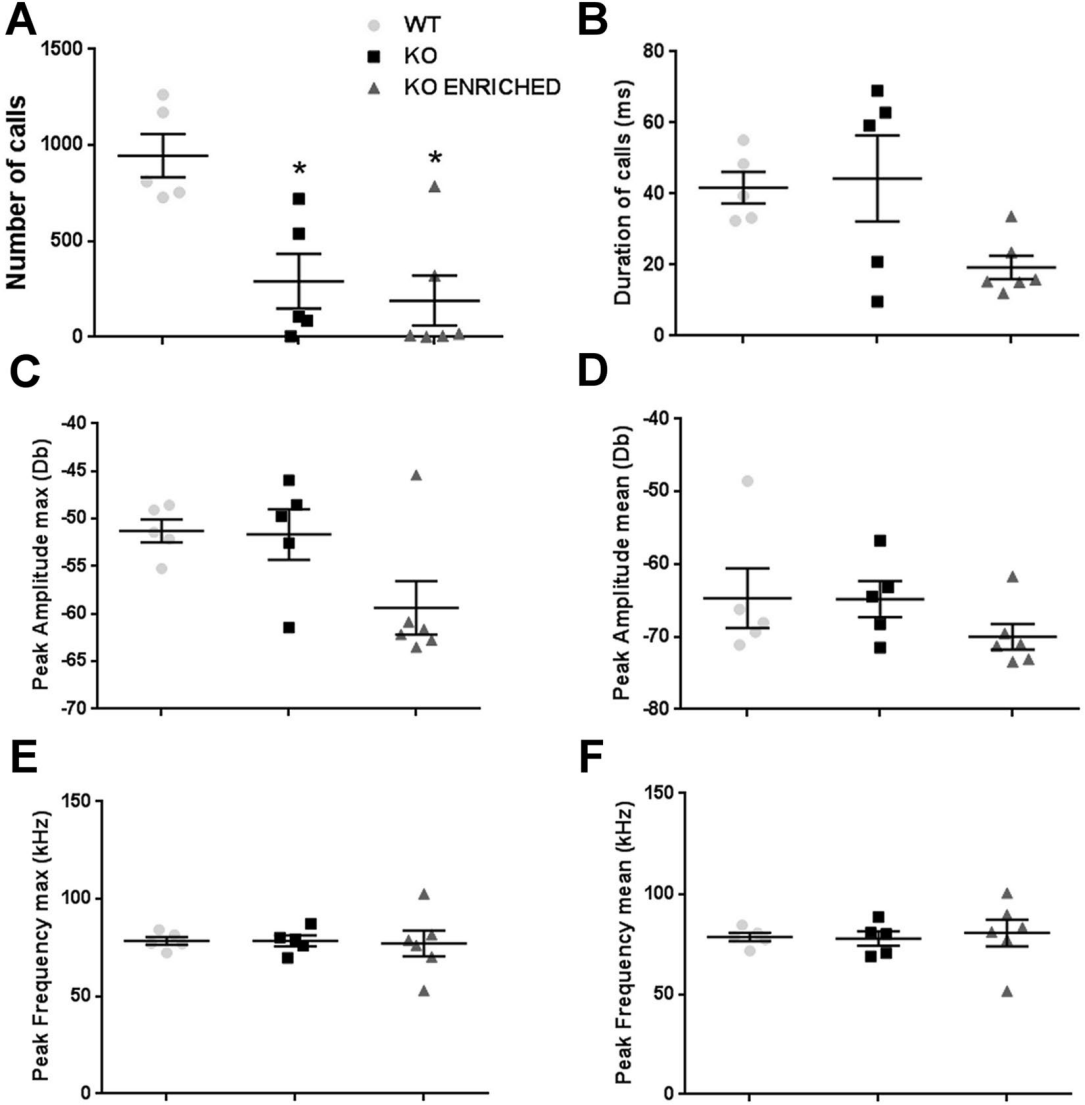
Quantitative analysis of ultrasonic communication in WT, KO and KO ENRICHED adults. (**A**) Number, (**B**) duration, (**C**) peak amplitude max, (**D**) peak amplitude mean, (**E**) peak frequency max and (**F**) peak frequency mean of USVs emitted by adults during male-female social interaction test. Data are presented as mean ± S.E.M. N = 5 couples of WT, 6 couples of KO and 6 couples of KO ENRICHED. *p < 0.05 for KO and KO ENRICHED vs WT mice (One-way ANOVA, followed by Sidak’s post-test analysis).

**Figure 6:**
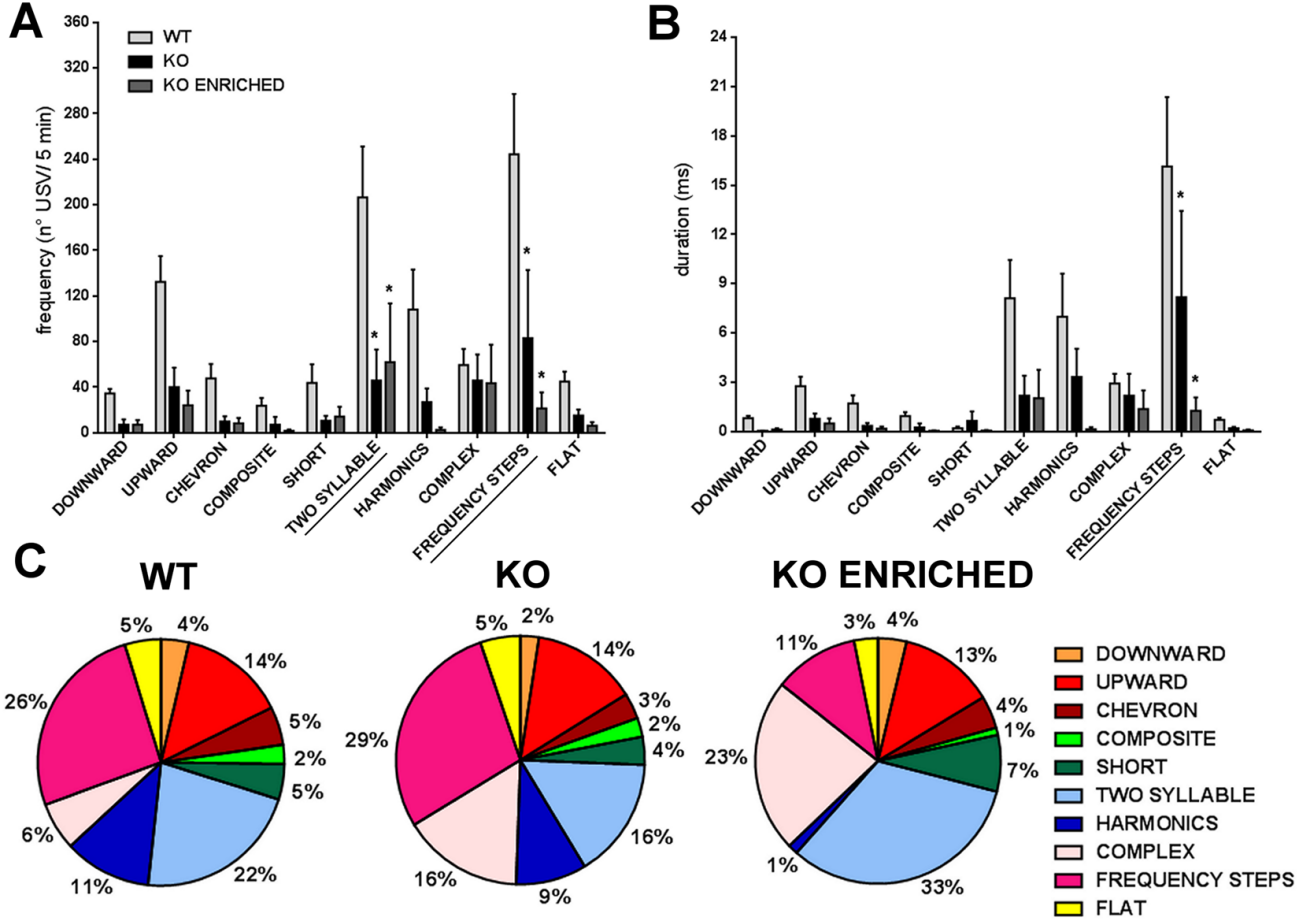
Qualitative analysis of ultrasonic communication in WT, KO and KO ENRICHED adults. (**A**) Number and (**B**) duration of different calls categories of WT, KO and KO ENRICHED adult mice. Data are presented as mean ± S.E.M. N = 5 couples of WT, 6 couples of KO and 6 couples of KO ENRICHED. *p < 0.05 for KO and KO ENRICHED vs WT mice (Two-way ANOVA, followed by Sidak’s post-test analysis). (**C**) Proportion of different call typologies for genotype expressed as the percentages in pie graphs.

During this test, USVs emitted by adult male interacting with oestrous female were recorded. Mice not vocalizing were excluded (2 WT, 1 KO and 1 KO enriched). KO mice emitted a lower number of USVs than WT [F (2, 13) = 9.832, p < 0.01; Fig. 5A]. USVs number of KO enriched was similar to those of KO mice. No significant differences in other vocalization traits were found (Fig. 5B–F). On the contrary, a qualitative analysis of ultrasonic communication revealed important differences between genotypes. In particular KO adult mice emitted a lower number of *two syllable* and *frequency steps* calls [F (18, 117) = 3.183, p < 0.0001; Fig. 6A] with a shorter duration of this last category than WT mice [F (18, 117) = 3.264, p < 0.0001; Fig. 6B]. KO enriched mice had an ultrasonic communication pattern similar to those of KO mice. Finally, Fig. 6C displays the vocal repertoire of mice represented as the proportions of different call typologies for genotype.

## Discussion

p50 KO is an inbred strain of mice that, as we previously demonstrated, presents cortical structural alterations, hyperactivity and social interaction deficit typical of NDDs animal models^42,43^. Now we analysed ultrasonic communication in p50 KO mice, both in pups and adults, and compared it with USVs of WT mice. Concerning maternal separation-induced USVs, we found that WT pups emitted an inverted U-shaped call emission pattern that followed a typical ontogenetic profile of USVs mouse pups^12,44^ and it was exacerbated in p50 KO pups. p50 KO pups emitted significantly more and longer USVs than WT pups. A detailed analysis displayed that WT pups emitted a quite homogenous repertoire of calls; on the other hand p50 KO pups emitted a different repertoire of calls, which included higher number of *two syllable* and *frequency steps* with a longer duration of these calls than WT pups.

Different studies have suggested the translational value of pups USVs, that can be compared to the human babies cry in the context of NDDs and ASD. This is an extensively studied theme and Scattoni, for first, proposed this analogy for the acoustic and functional features of USVs; they can reflect the same functions of babies cry such as their capacity to elicit parents attention and care and for their important role in social communication between mother and infant^14^. Other studies confirmed the idea that pups USVs can present a social communication form similar to the nonverbal infants calls^13,29^. Understanding USVs role in mice pups is not only necessary to understand the mother–pups interactions, but also to better know communication in children and this can have implications with the knowledge and early identification of NDDs, in particular of ASD^45^.

To further support the translational value of murine USVs study, a similar analysis of pups-mother communication has been applied also in humans, studying the crying of newborn babies^46^. Abnormal features of baby crying emerged to be strictly related to specific neurological disorders^47^, including also autism spectrum disorders^48,49^.

After the USVs analysis in pups, we focused our attention on USVs of adult mice. During male-female social interaction test, adult KO mice emitted decreased number of USVs than WT mice associated with a reduced social interaction, in particular reduced sniffing behaviours. We hypothesize that this reduced number of USVs found in adult p50 KO mice can be linked to reduced communication and social interaction typical of adult human patients with NDDs. But what is very interesting, is that the same categories of calls altered in KO pups were reduced in KO adult mice. Therefore, alterations of ultrasonic communication found in p50 KO mice are not generalized but call-specific; indeed only few types of calls are different in comparison to those of WT mice. This could have an important role in the context of specific call meaning.

Therefore, in p50 KO mice quantitative and qualitative alterations of ultrasonic communication were found. In other models of NDDs, similar altered calling patterns were detected. For instance, BTBR pups emitted more calls with a longer duration than their control mice^14^ and during adulthood, number of USVs of BTBR mice decreased^15^, as p50 KO mice. From a qualitative point of view, alterations of USVs were different but always call-typologies specific. Indeed, BTBR pups emitted an unusual pattern with a high number of *harmonics*, *two syllable* and *composite* calls^14^. In adulthood, during male-female social interaction test, BTBR emitted fewer *short* and *frequency steps* calls than control adult mice^15^. Furthermore, other call-typologies specific deficits are present in the literature, such as increased number of *frequency jump* calls on PND 7^50^, and decreased number of *downward* calls on PND 8 in Fmr1-KO compared to their WT pups^18^. Also at adulthood, during courtship Fmr1-KO mice emitted a higher proportion of *upward* syllables^19^, *complex, chevron* and *flat*, and a reduced number of *composite* and *frequency steps* than control mice^51^. In addition, as ultrasonic communication specific deficit, in another model of NDDs, Shank1^−/−^ mice, a categorical shift in the proportions of two clusters of frequency (first cluster between 50 and 80 kHz and second cluster between 80 and 100 kHz) was observed^22,52^. Interestingly, heterozygotes mice for 16p11.2 deletion displayed call-types specific alterations. These adult mice uttered calls in a different proportion for the three-phase male-female social interaction test. In particular, a higher percentage of *short* calls was found during first exposure to novel oestrus female (phase 1) and a lower percentage of *upward* calls after the female was removed from the interaction cage (phase 2). Also the number of calls in each category was different resulting in fewer *complex*, *two syllable*, *upward* and *frequency steps* in comparison with control mice in phase 1 and *two syllable* and *upward* in phase 2^28^.

Unfortunately, to date the meaning of mice USVs various typologies is unknown. In the future it will be compelling to understand the meaning, also because different mouse models of NDDs seem to have their own ultrasonic communication scheme.

We also wanted to investigate if ultrasonic communication of p50 KO mice could be influenced by maternal behaviour. One possible explanation of communication altered in p50 KO mice was linked to reduced maternal care observed in KO mothers. Indeed, KO mothers had a reduced frequency of maternal behaviours, such as nursing postures, in comparison with WT mothers.

In line with this hypothesis, the study of Kikusui and Hiroi suggested that poor maternal care can be considered a “self-generated environmental factor” of diseases like ASD, as it is induced, through atypical vocal sequences, by a genetic ASD risk carrier^53^. For instance, heterozygosity of Tbx1, an ASD risk gene, caused atypical pups USVs (less complex call types and less variable call sequences), which evoked less maternal behaviour^54^. Therefore, mutation of ASD risk genes can alter the neonatal call sequence, which renders pup’s social communication with mothers ineffective and maternal care less efficient^29,53,54^.

In this context, early social enrichment was performed to better understand the link between USVs and maternal behaviour. In particular, KO pups were exposed to early social enrichment (a WT female flanking their natural KO mother) until weaning and their USVs were recorded and analysed. First, we observed that KO enriched pups received more maternal care than standard KO pups, similar to those of WT pups. In addition, we analysed the individual contribution of the two females that took care of the pups to understand if increased nursing behaviour derived only by added WT female, or by changes in KO mother behaviour. We found that early social enrichment increased nursing behaviour for KO mothers. We evaluated also the effect of early social enrichment on WT pups, by adding a virgin WT female to the WT mother. We found that WT enriched pups received maternal care similar to WT pups.

Concerning developmental traits of pups, early social enrichment did not influence body weight of pups. Indeed, body weight of KO enriched did not significantly change compared to those of KO pups. In addition, some marks of delayed development were found in KO pups and only partially rescued in KO enriched pups. For example, KO pups did not display a significant preference between nest area and start area in the homing test contrary to WT and KO enriched pups. This may suggest a less developed olfactory discrimination for KO pups at the age of testing partially rescued by early social enrichment. Furthermore, KO pups had a delayed eyes opening compared to WT; on the contrary, KO enriched pups presented a heterogeneous phenotype, since most of them opened their eyes as at day 13 (as KO pups), but almost 40% opened eyes at day 12 (as WT pups). To date, no studies have reported that early social enrichment allows a recovery of these delayed development signals. It is known that enhanced social environment can regulate gene expression through epigenetic modulations but if it could influence developmental traits, such as eyes opening, is not well clarified yet. Future studies will help to understand this interesting theme.

Looking at quantitative and qualitative USVs analysis, we observed that USVs of KO enriched pups were very similar to those of standard KO pups. Adult KO enriched mice were hyperactive and presented social interaction deficit as KO adult mice. Their ultrasonic communication pattern was in line with KO mice.

In conclusion, from these analyses, it emerged that early social enrichment, despite able to modify some developmental traits in pups, had no effect on USVs number, duration and type, locomotor activities and social interaction in p50 KO pups and adults, strengthening the fact that, for p50 KO mice, genetic background prevails on environmental enrichment. On the contrary, in some models of NDDs, such as Fmr1-KO mice, early environmental enrichment through enhanced maternal stimulation, influenced ultrasound communication of pups, rescued the behavioural alterations shown in adulthood, such as hyperactivity and social deficit but not ultrasonic communication^32^. On this topic, we could hypothesize that the effect of p50 deletion is so strong on mice behaviour due to the importance of the NF-ĸB pathways for several basic processes. Indeed, NF-ĸB is one of the evolutionary conserved pathway, ubiquitously expressed, that regulates different vital functions such as inflammation, neurogenesis, cell plasticity and its genetic alterations cause severe behavioural impairments^41,42,55,56^.

## Methods

### Animals

Wild-type (B6;129PF2) and NF-κB p50 KO mice (B6;129P2-Nfkb 1tm 1 Bal/J) used for experiments were obtained in our animal facility from mating mice (Supplementary Methods). For early social enrichment experiment, double-mothering paradigm was applied^32^. In detail, NF-κB p50^−/−^ mouse (KO) male was replaced by a virgin nonlactating wild-type (WT) adult female in the pregnant KO female cage at late gestation until weaning of pups (KO enriched group). Instead, in the other conditions without social enrichment, WT and KO pregnant females were individually housed and they nursed alone their pups.

Different groups of mice were used in this study. First group of 17 adult primiparous mothers (4–6 month old) was evaluated for mother-pup interaction. Second group: offspring of other mothers kept behaviourally naïve, were tested for behavioural tests during infancy, adolescence and adulthood.

Animals were housed in standard cages in a 12 hours light/dark cycle (light phase: from 8:00 a.m. to 8:00 p.m.) with food and water available ad libitum. Temperature (22 °C) and humidity (50% ± 10) in the cage were automatically regulated by the Sealsafe Aero System by individually ventilated cages with EPA filters (Tecniplast Group, Italy). Mice were housed up to a maximum of 4 adults per cage or 1 mother with its litter or 1 female with 1 male (for mating). To reduce genetic variations, we used for all the experiments mice bred and tested in the same time lapse (4 months).

All experiments were performed in conformity with the European Communities Council Directive of 1986 (86/609/EEC), and approved by the Italian Ministry of Health, Animal care and use Committee of the University of Brescia.

### Mother–pup interaction

Maternal behaviour was analysed for 17 litters derived by 6 WT, 6 KO and 5 KO enriched mothers of first group. From PND 1 to 7, mothers were observed twice day for 1 h (at 11:00 a.m. and at 4:00 p.m.) using a method described by Oddi and colleagues^32^. Maternal behaviours, such as nursing postures, non nursing postures, pups grooming and nest building, were analysed and expressed as frequencies^57,58^.

### Developmental behavioural traits

Every day from PND 4 to 12, 37 WT (21 female and 16 male), 37 KO (19 female and 18 male) and 21 KO enriched (14 female and 7 male) pups of second group were weighed. Also their eyes opening was evaluated.

### Social recognition in the homing test

On PND 14, 37 WT (21 female and 16 male), 37 KO (19 female and 18 male) and 20 KO enriched (13 female and 7 male) pups of second group were assessed for social recognition in the homing test. After separation from the mother, each pup was transferred in a Plexiglas T-maze with three arms (10 × 8 cm), surrounded by a wall 15 cm high and 0.5 cm thick. Two arms of the apparatus named “start” and “clean” were covered by clean sawdust and another arm called “nest” with sawdust collected from the nest of the pup’s cage. The pup placed in the start arm was free to explore the maze for 5 min. Images captured by a camera above the maze, were analysed manually by the operator, measuring the first latency to reach the nest, the time spent in the nest, in clean and in start arms and locomotor activity by square crossing.

### Olfactory habituation/dishabituation

For the test of olfactory habituation/dishabituation adapted from^59^, 20 WT (10 female and 10 male), 20 KO (10 female and 10 male) and 17 KO enriched (10 female and 7 male) adolescents (6 weeks old) of second group were used. A sequential presentation of different odours was performed in three consecutive trials of 2 min duration each. Olfactory investigation such as nasal contact with the applicator (within a 2 cm distance) was evaluated (Supplementary Methods).

### Pups ultrasonic vocalizations

37 WT (21 female and 16 male), 37 KO (19 female and 18 male) and 21 KO enriched (14 female and 7 male) pups of second group were evaluated on PND 4, 6, 8 and 10 for USVs in response to maternal separation of 3 minutes, using Avisoft Bioacoustic system as previously described^16,32^, Supplementary Methods.

Spectrograms of each pup at PND 6 were inspected manually by the operator and ultrasonic vocalizations were classified into 10 categories, generally based on criteria previously described by Scattoni and colleagues^14^.

### Open field exploration test

Male WT, KO and KO enriched adult of the second group (4–6 month old, 6–7 mice for genotype) were introduced in the centre of the arena (40 × 40 cm in Plexiglas) and left free to explore for 5 minutes. A camera vertically montated 1.5 m above the arena recorded mice movements. Locomotor activity, expressed as total distance travelled, average speed and total time mobile, were automatically analysed with ANY-maze software.

### Male-female social interaction and adult ultrasonic vocalizations

For male-female social interaction, 7 couples of WT, KO and KO enriched adult mice of the second group were used. Male were isolated in their cages for 5 days and then an unfamiliar female of the same genotype was introduced in the cage of the isolated male for 5 minutes. On the day of test, the vaginal oestrous phase of females was checked as previously described^15,60^. Only females in oestrous phase were tested. Social behaviours (e.g. sniffing, following, mounting, contact and wrestling) and non social behaviours (e.g. self grooming and exploring) were recorded with a camera placed facing the side of the cage and analysed with Observer XT (version 14.1, Noldus, The Netherlands).

An ultrasound microphone suspended 20 cm above the cage, recorded USVs emitted by the male resident during 5 minutes interaction with oestrous female. Male-female social interaction induced USVs were quantitative analysed and manually classified as previously described for pups USVs.

### Statistical analysis

Statistical analysis was performed by GraphPad Prism 6 software (GraphPad, San Diego, California). For mother-pup interaction, homing test, olfactory habituation/dishabituation test, quantitative and qualitative USVs analysis of pups, qualitative USVs analysis of adults and detailed evaluation of social/non social behaviors, Two-way analysis of variance (ANOVA) followed by Tukey or Sidak’s multiple comparison test were used. For other behavioural tests, One-way ANOVA followed by Tukey or Sidak or Dunnet’s multiple comparison test was used. Data are presented as the means ± S.E.M., with the statistical significance level set at p < 0.05 (WT versus KO mice and KO versus KO enriched).

## Supplementary information

Supplementary Information

## Data Availability

All authors declare that the materials and data presented in this manuscript are available to readers.

## References

[1] Zippelius HM, Schleidt WM (1956). Ultraschall-laute bei jungen mausen (Ultrasonic vocalization in infant mice). Naturwissenschaften..

[2] D’Amato FR, Scalera E, Sarli C, Moles A (2005). Pups call, mothers rush: does maternal responsiveness affect the amount of ultrasonic vocalizations in mouse pups?. Behav Genet..

[3] Scattoni, M. L. & Branchi, I. Vocal repertoire in mouse pups: strain differences in *Handbook of Mammalian Vocalization* (ed. Brudzinsky, S. M.) 88–96 (Oxford, Academic Press, 2010).

[4] Holy TE, Guo Z (2005). Ultrasonic songs of male mice. PLoS Biol..

[5] Panksepp JB (2007). Affiliative behavior, ultrasonic communication and social reward are influenced by genetic variation in adolescent mice. PLoS One..

[6] Blumberg MS, Alberts JR (1990). Ultrasonic vocalizations by rat pups in the cold: an acoustic by-product of laryngeal braking?. Behav Neurosci..

[7] Blumberg MS, Sokoloff G (2001). Do infant rats cry?. Psychol Rev..

[8] Wöhr M, Schwarting RK (2013). Affective communication in rodents: ultrasonic vocalizations as a tool for research on emotion and motivation. Cell Tissue Res..

[9] Wöhr M, Scattoni ML (2013). Behavioural methods used in rodent models of autism spectrum disorders: current standards and new developments. Behav Brain Res..

[10] Lahvis GP, Alleva E, Scattoni ML (2011). Translating mouse vocalizations: prosody and frequency modulation. Genes Brain Behav..

[11] Fischer J, Hammerschmidt K (2011). Ultrasonic vocalizations in mouse models for speech and socio-cognitive disorders: insights into the evolution of vocal communication. Genes Brain Behav..

[12] Branchi I, Santucci D, Alleva E (2001). Ultrasonic vocalisation emitted by infant rodents: a tool for assessment of neurobehavioural development. Behav Brain Res..

[13] Scattoni ML, Crawley J, Ricceri L (2009). Ultrasonic vocalizations: a tool for behavioural phenotyping of mouse models of neurodevelopmental disorders. Neurosci Biobehav Rev..

[14] Scattoni ML, Gandhy SU, Ricceri L, Crawley JN (2008). Unusual repertoire of vocalizations in the BTBR T+tf/J mouse model of autism. PLoS One..

[15] Scattoni ML, Ricceri L, Crawley JN (2011). Unusual repertoire of vocalizations in adult BTBR T+tf/J mice during three types of social encounters. Genes Brain Behav..

[16] Gaudissard J (2017). Behavioral abnormalities in the Fmr1-KO2 mouse model of fragile X syndrome: The relevance of early life phases. Autism Res..

[17] Gauducheau M (2017). Age-specific autistic-like behaviors in heterozygous Fmr1-KO female mice. Autism Res..

[18] Roy S, Watkins N, Heck D (2012). Comprehensive analysis of ultrasonic vocalizations in a mouse model of fragile X syndrome reveals limited, call type specific deficits. PLoS One..

[19] Belagodu AP, Johnson AM, Galvez R (2016). Characterization of ultrasonic vocalizations of Fragile X mice. Behav Brain Res..

[20] Sungur AÖ, Schwarting RKW, Wöhr M (2018). Behavioral phenotypes and neurobiological mechanisms in the Shank1 mouse model for autism spectrum disorder: A translational perspective. Behav Brain Res..

[21] Wöhr M (2014). Ultrasonic vocalizations in Shank mouse models for autism spectrum disorders: detailed spectrographic analyses and developmental profiles. Neurosci Biobehav Rev..

[22] Sungur AÖ, Schwarting RK, Wöhr M (2016). Early communication deficits in the Shank1 knockout mouse model for autism spectrum disorder: Developmental aspects and effects of social context. Autism Res..

[23] Peñagarikano O (2011). Absence of CNTNAP2 leads to epilepsy, neuronal migration abnormalities, and core autism-related deficits. Cell..

[24] Ju A (2014). Juvenile manifestation of ultrasound communication deficits in the neuroligin-4 null mutant mouse model of autism. Behav Brain Res..

[25] Wöhr M (2013). Developmental delays and reduced pup ultrasonic vocalizations but normal sociability in mice lacking the postsynaptic cell adhesion protein neuroligin2. Behav Brain Res..

[26] Takayanagi Y (2005). Pervasive social deficits, but normal parturition, in oxytocin receptor-deficient mice. Proc Natl Acad Sci USA.

[27] Mosienko V, Beis D, Alenina N, Wöhr M (2015). Reduced isolation-induced pup ultrasonic communication in mouse pups lacking brain serotonin. Mol Autism..

[28] Yang M (2015). 16p11.2 Deletion Syndrome Mice Display Sensory and Ultrasonic Vocalization Deficits During Social Interactions. Autism Res..

[29] Esposito G, Hiroi N, Scattoni ML (2017). Cry, baby, cry: Expression of Distress as a Biomarker and Modulator in Autism Spectrum Disorder. Int J Neuropsychopharmacol..

[30] Renner, M. J. & Rosenzweig, M. R. Enriched and Impoverished Environments. *Effects on* Brain *and Behavior* (eds Renner, M. J. & Rosenzweig, M. R.) (Springer, 1987).

[31] D’Amato FR (2011). Intensification of maternal care by double-mothering boosts cognitive function and hippocampal morphology in the adult offspring. Hippocampus..

[32] Oddi D (2015). Early social enrichment rescues adult behavioral and brain abnormalities in a mouse model of fragile X syndrome. Neuropsychopharmacology..

[33] Pereira SG, Oakley F (2008). Nuclear factor-kappaB1: regulation and function. Int J Biochem Cell Biol..

[34] Zhang Yonggang, Hu Wenhui (2012). NFκB signaling regulates embryonic and adult neurogenesis. Frontiers in Biology.

[35] Boersma MC (2011). A requirement for nuclear factor-kappaB in developmental and plasticity-associated synaptogenesis. J Neurosci..

[36] Bonini SA (2011). Nuclear factor κB-dependent neurite remodeling is mediated by Notch pathway. J Neurosci..

[37] Mattson MP, Meffert MK (2006). Roles for NF-kappaB in nerve cell survival, plasticity, and disease. Cell Death Differ..

[38] Bortolotto V, Cuccurazzu B, Canonico PL, Grilli M (2014). NF-κB mediated regulation of adult hippocampal neurogenesis: relevance to mood disorders and antidepressant activity. Biomed Res Int..

[39] Gutierrez H, Davies AM (2011). Regulation of neural process growth, elaboration and structural plasticity by NF-κB. Trends Neurosci..

[40] Kassed CA, Herkenham M (2004). NF-kappaB p50-deficient mice show reduced anxiety-like behaviors in tests of exploratory drive and anxiety. Behav Brain Res..

[41] Denis-Donini S (2008). Impaired adult neurogenesis associated with short-term memory defects in NF-kappaB p50-deficient mice. J Neurosci..

[42] Bonini SA (2016). Cortical Structure Alterations and Social Behavior Impairment in p50-Deficient Mice. Cereb Cortex..

[43] Mastinu A (2018). Melanocortin 4 receptor stimulation improves social deficits in mice through oxytocin pathway. Neuropharmacology..

[44] Rosenblatt, J. S. & Lehrman, D. S. Maternal behavior in the laboratory rat in *Maternal behavior in mammals* (ed. Rheingold, H. L.) 8-57 (John Wiley & Sons, 1963).

[45] Ashbrook DG (2018). Born to Cry: A Genetic Dissection of Infant Vocalization. Front Behav Neurosci..

[46] Orlandi S, Manfredi C, Bocchi L, Scattoni ML (2012). Automatic newborn cry analysis: a non-invasive tool to help autism early diagnosis. Conf Proc IEEE Eng Med Biol Soc..

[47] Michelsson K, Michelsson O (1999). Phonation in the newborn, infant cry. Int J Pediatr Otorhinolaryngol..

[48] Venuti P, Esposito G, Giusti Z (2004). A qualitative analysis of crying and vocal distress in children with autism. Journal of Intellectual Disability Research..

[49] Orlandi, S. *et al*. Study of cry patterns in infants at high risk for autism. *ISCA Archive*, http://www.isca-speech.org/archive/maveba_2011 (2011).

[50] Lai JK (2014). Temporal and spectral differences in the ultrasonic vocalizations of fragile X knock out mice during postnatal development. Behav Brain Res..

[51] Hodges SL, Nolan SO, Reynolds CD, Lugo JN (2017). Spectral and temporal properties of calls reveal deficits in ultrasonic vocalizations of adult Fmr1 knockout mice. Behav Brain Res..

[52] Wöhr M, Roullet FI, Hung AY, Sheng M, Crawley JN (2011). Communication impairments in mice lacking Shank1: reduced levels of ultrasonic vocalizations and scent marking behavior. PLoS One..

[53] Kikusui T, Hiroi N (2017). A Self-Generated Environmental Factor as a Potential Contributor to Atypical Early Social Communication in Autism. Neuropsychopharmacology..

[54] Takahashi T (2016). Structure and function of neonatal social communication in a genetic mouse model of autism. Mol Psychiatry..

[55] Grilli M, Memo M (1999). Nuclear factor-kappaB/Rel proteins: a point of convergence of signalling pathways relevant in neuronal function and dysfunction. Biochem Pharmacol..

[56] Kaltschmidt B, Kaltschmidt C (2009). NF-kappaB in the nervous system. Cold Spring Harb Perspect Biol..

[57] Champagne FA, Curley JP, Keverne EB, Bateson PP (2007). Natural variations in postpartum maternal care in inbred and outbred mice. Physiol Behav..

[58] Curley JP, Jensen CL, Franks B, Champagne FA (2012). Variation in maternal and anxiety-like behavior associated with discrete patterns of oxytocin and vasopressin 1a receptor density in the lateral septum. Horm Behav..

[59] Yang, M. & Crawley, J. N. Simple behavioral assessment of mouse olfaction in *Current Protocols in Neuroscience* (ed. John Wiley & Sons). 8.24.1–8.24.12 (2009). 10.1002/0471142301.ns0824s48 PMC2753229 19575474

[60] Caligioni, C. S. Assessing reproductive status/stages in mice in *Current Protocols in Neuroscience* (ed. John Wiley & Sons) A.4I.1-A.4I.8 (2009). 10.1002/0471142301.nsa04is48 PMC2755182 19575469

