# Supplementary material for: Specific profile of ultrasonic communication in a mouse model of neurodevelopmental disorders
Source: Sci Rep. 2019 Nov 4;9:15912. doi: 10.1038/s41598-019-52378-0 (PMC6828716; doi:10.1038/s41598-019-52378-0)
Supplement: Supplementary file 1 — Supplementary Information [file 41598_2019_52378_MOESM1_ESM.docx]

**Specific profile of ultrasonic communication in a mouse model of neurodevelopmental disorders**

Marika Premoli, Sara Anna Bonini, Andrea Mastinu, Giuseppina Maccarinelli, Francesca Aria, Giulia Paiardi, Maurizio Memo

**SUPPLEMENTARY METHODS**

**Animals**

Each breeder adult male mouse was maintained in the same cage with an adult virgin female for mating. At late gestation (from day 15 after fecundation) the female was individually housed and inspected twice daily at 10:00 a.m. and 6:00 p.m. to determinate the day of pups’ birth (postnatal day, PND 0).

**Olfactory habituation/dishabituation**

First, the mouse was placed in the testing cage for habituation with one applicator put into a hole of the grid cage. After 30 min, a 10 µl drop of orange extract (Flora, Italy; 10^-6^ dilution) was placed on the cotton-tip part of the applicator and it was transferred in the home cage for 2 min. This procedure was repeated twice with 1-min intervals. Then a new applicator containing 10 µl drop of vanilla extract (Flora, Italy; 10^-6^ dilution) was placed in the subject’s cage for 2 min and another two trials were performed. Finally, another cotton-tipped swab saturated with social odours (a mixture of urine from unfamiliar same gender or opposite gender WT mice) was introduced in the mouse cage for three times.

**Pups ultrasonic vocalizations**

Each pup was placed into a glass container (diameter 5 cm, height 10 cm) with fresh clean bedding and its vocalizations were recorded for 3 minutes with an ultrasound microphone (Condenser ultrasound microphone CM16/CMPA, Avisoft Bioacoustics, Berlin, Germany) sensitive to frequencies of 10-180 kHz, placed 20 cm above the container.

The microphone was connected via an external audio interface (USG 116, Avisoft Bioacoustics, Berlin, Germany) to a personal computer, where acoustic data were displayed in real time by Avisoft Recorder (version 4.2; Avisoft Bioacoustics), and vocalizations were recorded with a sampling rate of 250 kHz in16 bit format [24]. Using SasLab Pro (version 5.2; Avisoft Bioacoustics), sound files were analysed and a fast Fourier transformation (FFT) was conducted (512 FFT-length, 100% frame, Hamming window and 75% time window overlap). Spectrograms were generated at frequency resolution of 488 Hz and a time resolution of 0.512 ms. Signals below 40 kHz were cut to reduce background noise to 0 dB. For USVs detection, an automatic threshold-based algorithm and a hold-time mechanism (hold time: 10 ms) were used. Vocalization traits analysed were number and duration of calls together with the peak frequency max, peak frequency mean, peak amplitude max and peak amplitude mean.

At the end of the 3 minutes recording session, each pup was weighed and its abdominal temperature measured with a digital infrared thermometer (Corman, Italia). Pups were marked on the paw with animal tattoo ink on PND 4, immediately after behavioural testing.

**Statistical analysis**

Statistical analysis was performed by GraphPad Prism 6 software (GraphPad, San Diego, California). Two-way analysis of variance (ANOVA) followed by Tukey or Sidak’s multiple comparison test or One-way ANOVA followed by Tukey’s multiple comparison test were used for all tests. Data are presented as the means ± S.E.M., with the statistical significance level set at p<0.05 (WT versus KO mice and KO versus KO enriched).

**SUPPLEMENTARY FIGURE**

**
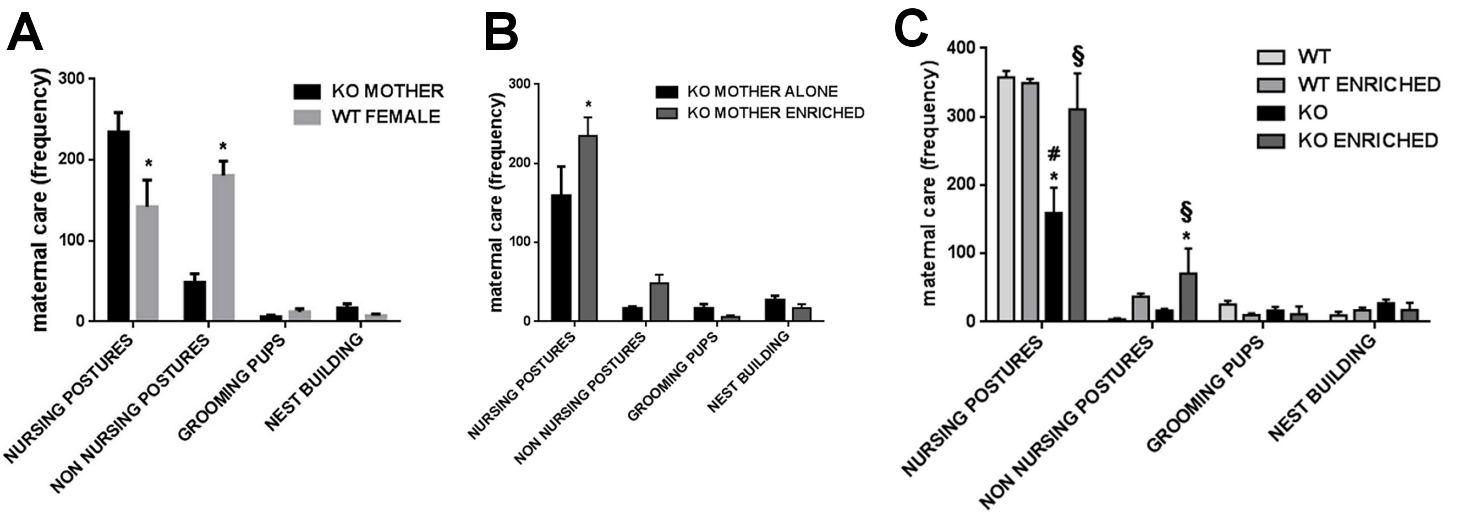
Supplementary Fig. S1.** **Maternal care analysis**. (A) Representation of maternal behaviours frequency of KO mothers and WT females during early social enrichment experiment. Data are presented ad mean ± S.E.M. N = 5 KO MOTHERS and 5 WT FEMALES. *p < 0.05 for KO MOTHERS vs WT FEMALES. For statistical analysis, Two-way ANOVA followed by the Sidak’s post-test was used. (B) Maternal care frequency of KO mother when they were alone and KO mothers in enrichment context. Data are presented ad mean ± S.E.M. N = 6 KO MOTHERS ALONE and 5 KO MOTHERS ENRICHED. *p < 0.05 for KO MOTHERS ENRICHED vs KO MOTHERS ALONE. For statistical analysis, Two-way ANOVA followed by the Sidak’s post-test was used. (C) Maternal activities analysis of WT, WT enriched (WT mother with virgin nonlactating WT female), KO and KO enriched females. Data are presented as means ± S.E.M. N = 6 WT, 6 WT ENRICHED, 6 KO and 5 KO ENRICHED. *p < 0.05 for KO and KO ENRICHED vs WT; §p < 0.05 for KO ENRICHED vs KO and #p < 0.05 for KO vs WT ENRICHED. For statistical analysis, Two-way ANOVA, followed by Tukey’s post-test was used.

**
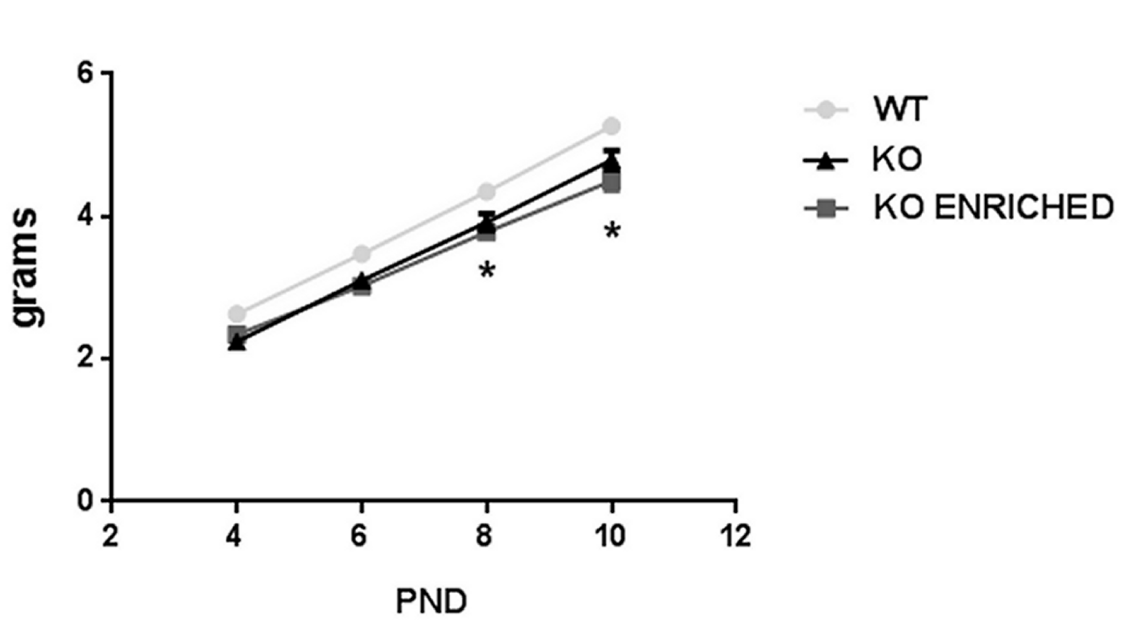
**

**Supplementary Fig. S2.** **Body weight of WT, KO and KO enriched pups.** Body weight of pups was measured from PND 4 to 10.

Data are presented ad mean ± S.E.M. N = 37 WT, 37 KO and 21 KO ENRICHED. *p < 0.05 for KO and KO ENRICHED vs WT. For statistical analysis, Two-way ANOVA followed by the Sidak’s post-test was used.

**
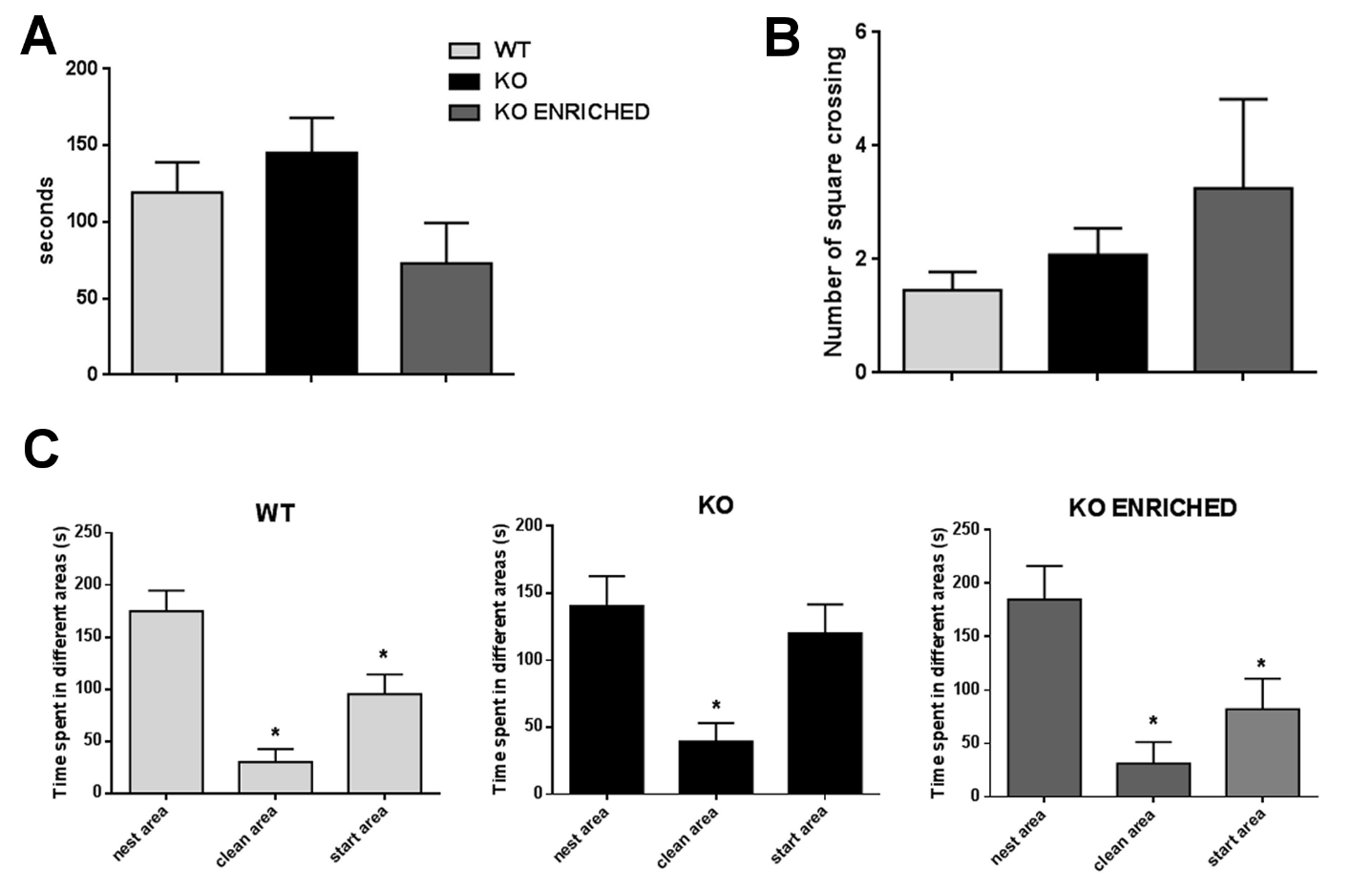
**

**Supplementary Fig. S3.** **Social recognition analysis in WT, KO and KO enriched pups.** Results of the homing test expressed as (A) the latency in seconds to reach the nest area and (B) locomotor activity by crossing of different areas. Data are presented ad mean ± S.E.M. N = 37 WT, 37 KO and 20 KO ENRICHED. For statistical analysis, Two-way ANOVA followed by the Sidak’s post-test was used. (C) Time in seconds spent in all different areas for all genotypes. Data are presented ad mean ± S.E.M. N = 37 WT, 37 KO and 20 KO ENRICHED. *p < 0.05 for time spent in the clean and start area vs nest area for WT and KO ENRICHED; *p < 0.5 for time spent in the clean vs nest area for KO. For statistical analysis, One-way ANOVA followed by the Tukey’s post-test was used.


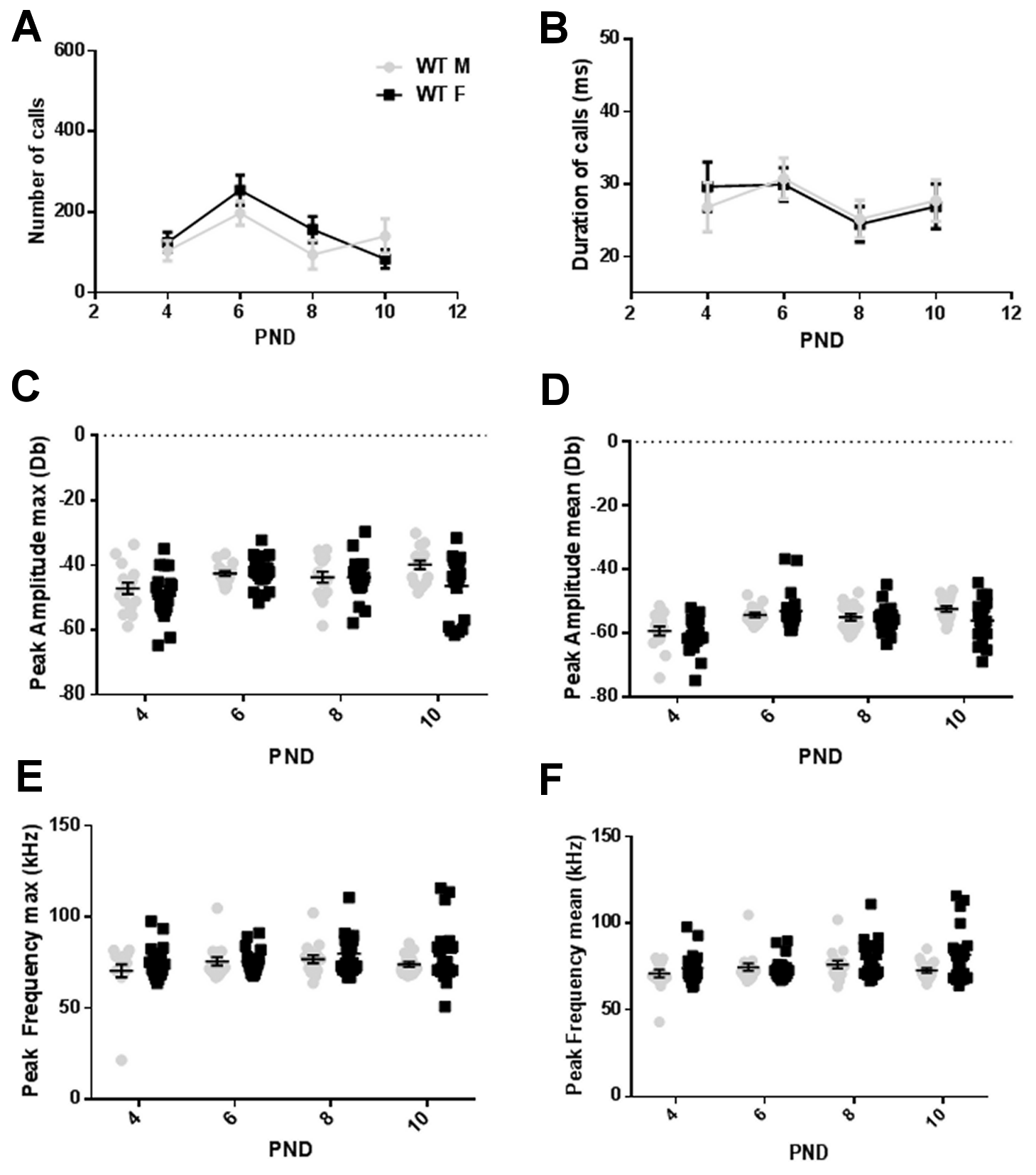


**Supplementary Fig. S4. Differences in patterns of calling between female and male WT pups. (A)** Number, (B) duration, (C) peak amplitude max, (D) peak amplitude mean, (E) peak frequency max and (F) peak frequency mean of vocalizations emitted from male and female WT pups from PND 4 to 10.

Data are presented ad mean ± S.E.M. N = 21 female and 16 male. For statistical analysis, Two-way ANOVA followed by the Sidak’s post-test was used.


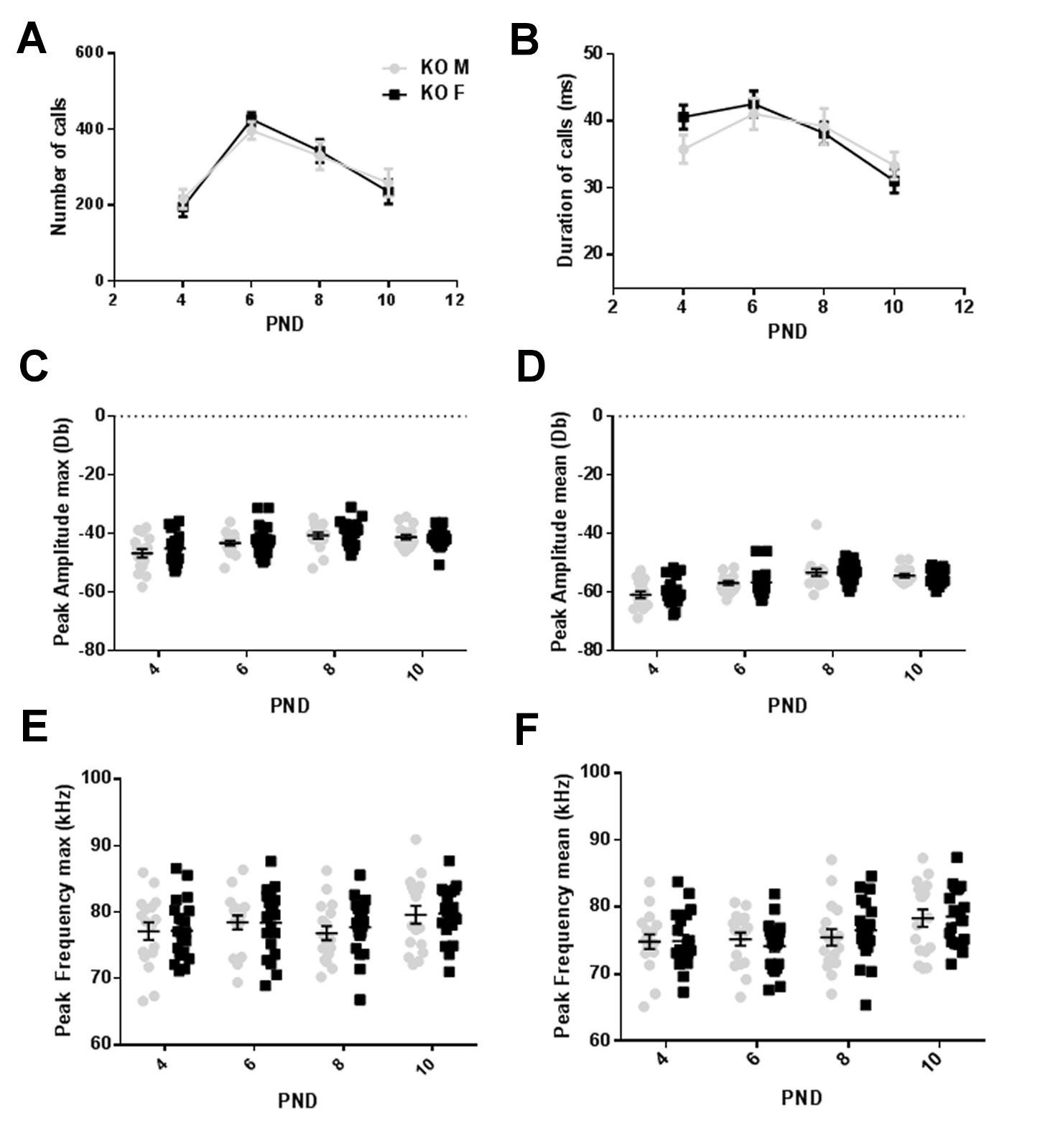


**Supplementary Fig. S5. Differences in patterns of calling between female and male KO pups. (A)** Number, (B) duration, (C) peak amplitude max, (D) peak amplitude mean, (E) peak frequency max and (F) peak frequency mean of vocalizations emitted from male and female KO pups from PND 4 to 10.

Data are presented ad mean ± S.E.M. N = 19 female and 18 male. For statistical analysis, Two-way ANOVA followed by the Sidak’s post-test was used.


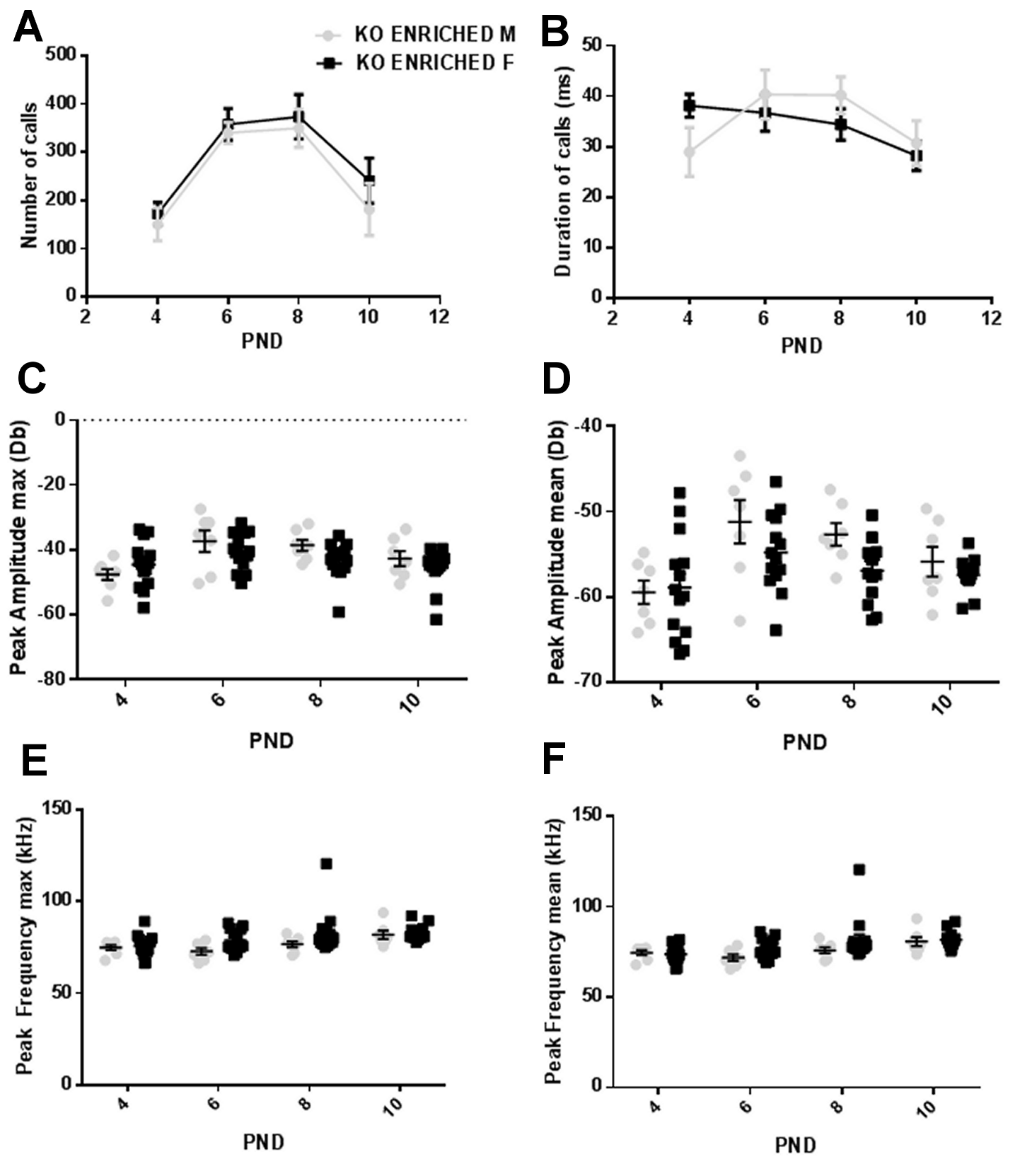


**Supplementary Fig. S6. Differences in patterns of calling between female and male KO ENRICHED pups.** (A) Number, (B) duration, (C) peak amplitude max, (D) peak amplitude mean, (E) peak frequency max and (F) peak frequency mean of vocalizations emitted from male and female KO ENRICHED pups from PND 4 to 10.

Data are presented ad mean ± S.E.M. N = 14 female and 7 male. For statistical analysis, Two-way ANOVA followed by the Sidak’s post-test was used.

**
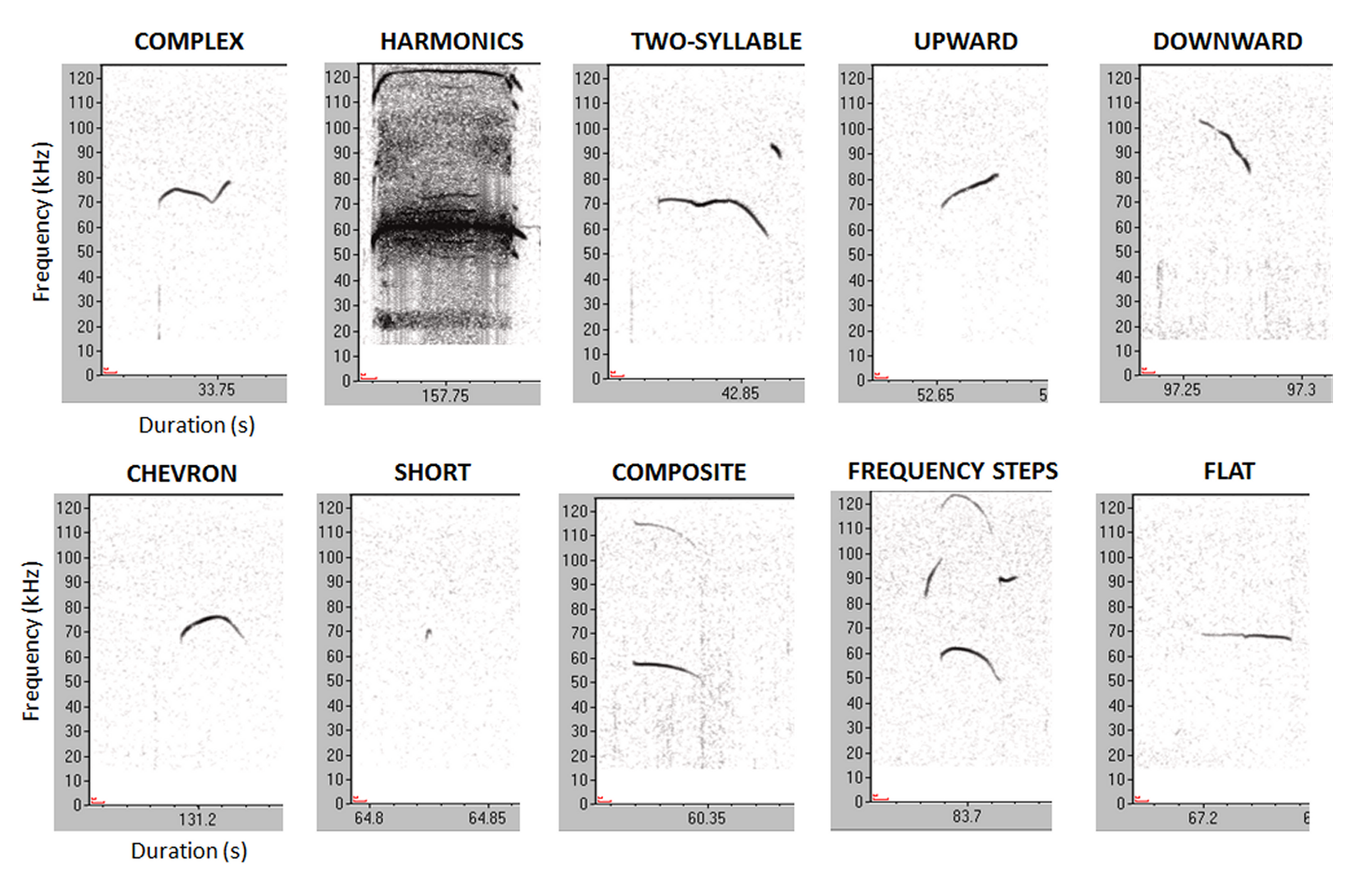
Supplementary Fig. S7. Examples of calls classification.** Typical sonograms of USVs classified into different calls categories emitted by WT pups.
